# Maternal gestational diabetes in singleton pregnancies conceived by ART may be modified by periconceptional B vitamins

**DOI:** 10.3389/fnut.2022.1069911

**Published:** 2023-01-18

**Authors:** Minyu Li, Yanping Chen, Yongxiang Wang, Hong Wang, Xueteng Ding, Guoju Li

**Affiliations:** ^1^Public Health School, Medical College of Qingdao University, Qingdao, Shandong, China; ^2^Qingdao Women and Children’s Hospital, Qingdao University, Qingdao, Shandong, China

**Keywords:** ART, gestational diabetes, B vitamins, blood glucose, interaction

## Abstract

**Background:**

The risk of maternal gestational diabetes mellitus (GDM) may be influenced by pregnancies conceived through assisted reproductive technology (ART). However, the influence of the dosage of B vitamins (folate, vitamin B6 and vitamin B12) on GDM weren’t considered. Thus, we hypothesized that periconceptional B vitamins could modify maternal GDM in singleton pregnancies conceived by ART.

**Methods:**

This study is a prospective cohort study using data from 3,252 women with singleton pregnancies and received a 75 g oral glucose tolerance test (OGTT) at 24–28 weeks of gestation. We included an interaction term in the multivariable logistic and linear regression models, respectively, to test our hypothesis.

**Results:**

Women who underwent ART were significantly associated with the incidence of GDM compared with spontaneous pregnancy women. The adjusted odds ratio (aOR) was 1.59, and the 95% confidence interval (CI) was 1.08–2.34. ART pregnancies also elevated OGTT (oral glucose tolerance test) 1-h blood glucose levels and OGTT 2-h blood glucose levels (*P* < 0.05). A positive association between dietary vitamin B6 (aOR = 1.60, 95% CI: 1.13–2.27), dietary vitamin B12 (aOR = 1.88, 95% CI: 1.34–2.64) and dietary folate (aOR = 1.66, 95% CI: 1.19–2.32) with GDM risk comparing the highest to the lowest quartile (all *P*_*trend*_ < 0.001). The aORs of GDM for inadequate (< 400 μg/day), adequate (400–800 μg/day), and excessive (> 800 μg/day) supplemental folate intake were 1.00, 0.93, and 1.30, respectively (*P*_*trend*_ = 0.033). Since only the supplemental folate illustrates a statistically significant interaction with ART (*P* for interaction < 0.05), the association between ART and GDM and OGTT blood glucose levels stratifying by supplemental folate were further evaluated. These increased risks of GDM (aOR = 1.62, 95% CI: 1.39–3.39) and the regression coefficients (β) of 1-h blood glucose (β = 0.76, 95% CI: 0.39–1.13) and 2-h blood glucose (β = 0.60, 95% CI: 0.29–0.92) in the multiple linear regression model were significant only in the ART group with excessive supplemental folate (> 800 μg/day).

**Conclusion:**

The risk of GDM is significantly elevated, particularly among those women who conceived ART with the intake of excessive supplemental folate (> 800 μg/day).

## Introduction

The number of pregnancies through assisted reproductive technology (ART) has been on the rise significantly since the mid-1980s when *in vitro* fertilization (IVF) became available in China (1). Following this, it is important to check the possible dangers to mothers and their offspring. Numerous studies have found that compared to women who conceive spontaneously, women who conceive though IVF appear at a higher risk of obstetric and perinatal complications (2–5). One of the most common and important pregnancy complications is gestational diabetes mellitus (GDM). The latest systematic review and meta-analysis showed a higher risk of GDM in women whose pregnancy is through ART than spontaneous (6). However, the study on the relationship between ART and OGTT blood glucose levels remains to be examined.

Maternal advanced age, obesity, multiple pregnancies, and polycystic ovary syndrome (PCOS) are the major risk factors for GDM. They are also frequently present in women who received ART, suggesting a potential association between GDM and ART (7–10). Some modifiable risk factors during pregnancy, in addition to BMI, include vitamin supplements. B vitamins are necessary for the One Carbon Cycle (C1) and are crucial for numerous metabolic pathways and methylation processes. Folate interacts closely with vitamin B6 and B12 for functional effects on the C1 network (11). Meanwhile, folate and vitamin B6 and B12 are crucial enzymatic cofactors in the synthesis of methionine from homocysteine (12, 13), and their lacking increases homocysteine concentrations in the blood (14, 15). Since elevated homocysteine is associated with insulin resistance, it leads to cause the risk of GDM (16, 17). Hence, our study investigated the effect for folate and vitamin B6 and B12 on GDM.

Although the previous study showed that folate supplementation and vitamin B12 were related to a higher probability of live birth among women who underwent ART (18, 19), the combined effect of B vitamins (folate, vitamin B6, and vitamin B12) and women who received ART on GDM has not been investigated till now. To address this gap, this study aimed to determine whether women who underwent ART are an independent risk factor for the development of GDM from a prospective cohort study. Moreover, it is further hypothesized that maternal B vitamins (folate, vitamin B6, and vitamin B12) intake from pre-pregnancy to the first trimester would alter the relationship between ART and GDM and maternal blood glucose levels.

## Materials and methods

### Study population and data sources

On May 1, 2019, a progressive prospective cohort study of pregnant women between 4 to 14 weeks was initiated. The data reported in this paper are as of February 21, 2022. A total of 3,980 pregnant women were recruited, and the details of this study have been described previously (20). All of the pregnant women in this study were part of the Qingdao Women and Children Hospital Health Cohort, a prospective cohort aimed at determining the impact of maternal dietary, environmental, and lifestyle exposures on the health of pregnant women and their children. During registration, questionnaire-based interviews were used to collect information on dietary information, social demographic status, reproductive variables, disease family history, the use of supplements, lifestyle factors, and illnesses. Throughout the follow-up visits during mid-pregnancy and late pregnancy, they provided information on lifestyle, dietary intake, and supplements.

**FIGURE 1:**
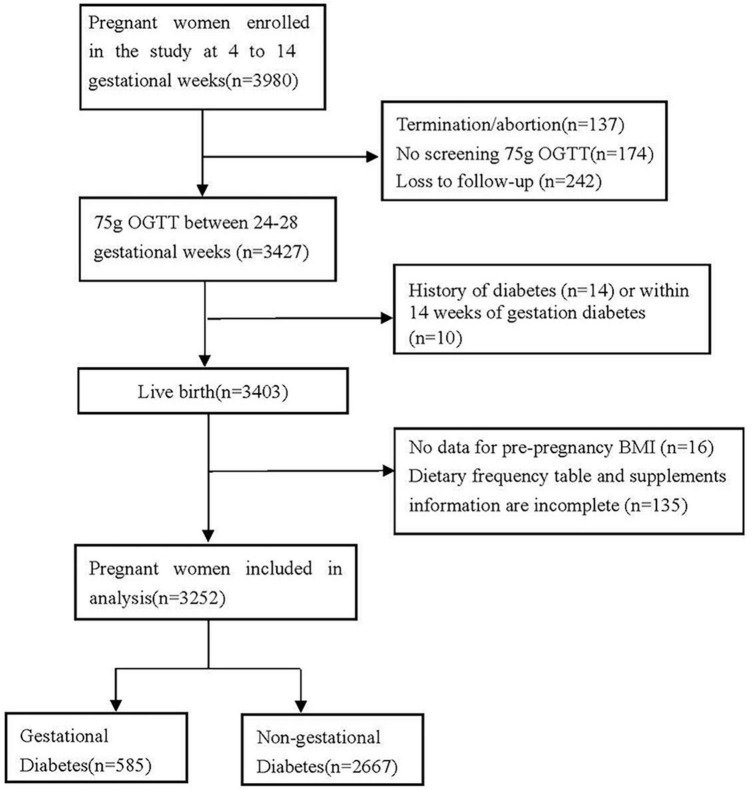
Flow chart of the screening process for the selection of eligible participants.

**FIGURE 2:**
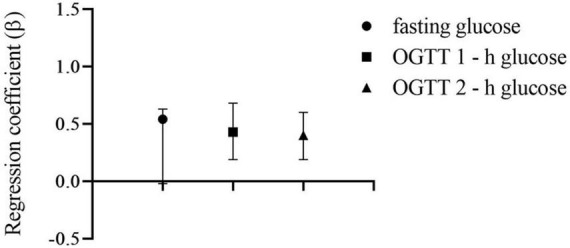
Association (regression coefficients ± 95% CIs) of ART with maternal OGTT glucose levels, reference to control group of women who conceived spontaneously. All models were adjusted education, occupation, smoking, passive smoking, age, pre-pregnancy BMI, abortion history, PCOS, GDM history, macrosomia, family history of diabetes and family history of gestational diabetes. Among all the 2,258 women of which 2,031 were spontaneous conception and 227 were receiving ART.

Pregnant women were included with detailed information on the doses and duration of B vitamins supplements, the way of conception, and height and weight before to pregnancy. Following are the exclusion criteria: (1) Termination or abortion (*n* = 137); (2) missing the follow-up (*n* = 242) and loss the information of 75 g OGTT screening (*n* = 174); (3) history of diabetes (*n* = 14) or within 14 weeks of gestation diabetes (i.e., pregnant women whose fasting blood glucose value was higher than 5.1 mmol/L on the day of questionnaire collection) (*n* = 10); (4) height and weight of pregnant women before pregnancy is incomplete or missing (*n* = 16); (5) Dietary and supplemental information are incomplete, with unclear doses and duration (*n* = 135) (Figure 1). A total of 3,252 singleton births were considered for the final analysis. Among all the women in this study, 2,258 had complete three OGTT blood glucose levels, of which 2,031 were spontaneous conception and 227 received ART. Thus, 2,258 were included in the analysis of blood glucose levels (Figure 2 and Table 5). Participation in the study was voluntary, and everyone was provided written informed consent.

**TABLE 1:** Demographic characteristics of women with spontaneous conception and ART.

Variables	Spontaneous conception (*n* = 2,919)	ART (*n* = 333)	*P*
Maternal age (years)			**<0.001**
<35	2,515 (86.16%)	249 (74.77%)	
≥35	404 (13.84%)	84 (25.23%)	
Educational level			**<0.001**
Secondary school or below	159 (5.45%)	43 (12.91%)	
High school	266 (9.11%)	48 (14.41%)	
College/university	2,053 (70.33%)	205 (61.56%)	
Postgraduate and above	441 (15.11%)	37 (11.11%)	
Occupation			**<0.001**
No	401 (13.74%)	94 (28.23%)	
Yes	2,518 (86.26%)	239 (71.77%)	
Parity			**<0.001**
Nulliparous	1,278 (57.39%)	168 (79.62%)	
Multiparous	949 (42.61%)	43 (20.38%)	
Previous GDM			0.058
No	1,910 (94.84%)	215 (97.73%)	
Yes	104 (5.16%)	5 (2.27%)	
Maternal smoking before or during pregnancy			0.711
No	2,896 (99.21%)	331 (99.40%)	
Yes	23 (0.79%)	2 (0.60%)	
Pre-pregnancy BMI group (kg/m^2^)			**0.002**
<24	2,176 (74.55%)	222 (66.67%)	
≥24	743 (25.45%)	111 (33.33%)	
Previous delivery of babies ≥ 4 kg			0.085
No	1,820 (95.04%)	209 (97.66%)	
Yes	95 (4.96%)	5 (2.34%)	
PCOS			**< 0.001**
No	2,263 (89.98%)	212 (73.10%)	
Yes	252 (10.02%)	78 (26.90%)	
Dietary B6 (mg/day)	1.06 (0.65–1.51)	1.01 (0.58–1.57)	0.673
Supplemental B6 (mg/day)	1.90 (0.10–2.60)	2.40 (0.41–2.60)	**0.001**
Total B6 (mg/day)	2.87 (1.47–3.73)	3.18 (1.91–3.85)	**0.019**
Dietary B12 (μg/day)	1.69 (0.63–2.99)	1.70 (0.67–3.24)	0.357
Supplemental B12 (μg/day)	4.00 (0.26–4.87)	4.00 (0.60–5.20)	**0.025**
Total B12 (μg/day)	4.78 (2.55–6.49)	5.04 (3.72–6.65)	**0.029**
Dietary folate (μg/day)	174.50 (85.38–282.55)	189.44 (83.41–293.50)	0.371
Supplemental folate (μg/day)	800 (350–1,540)	800 (400–1,600)	IQR
Total folate (μg/day)	843.05 (390.52–1404.96)	919.24 (527.11–1740.76)	**<0.001**
Fasting glucose (mmol/L)	4.53 (0.48)	4.64 (0.66)	0.120
OGTT 1-h glucose (mmol/L)	7.79 (1.61)	8.36 (1.86)	**<0.001**
OGTT 2-h glucose (mmol/L)	6.72 (1.32)	7.27 (1.60)	**<0.001**

Data are presented as n (%), mean ± SD or median (IQR). *P*-values are determined by chi-square, independent *t*-test or Mann–Whitney *U*-test. Bold values indicate statistically significant values.

**TABLE 2:** Association of ART with maternal gestational diabetes mellitus.

	*N* (%)	Crude OR (95% CI)	*P*-value (95% CI)	Adjusted OR^a^	*P* -value
Spontaneous conception	2,919 (89.76)	1.00		1.00	
ART	333 (10.24)	1.78 (1.37–2.31)	**<0.001**	**1.59 (1.08**–**2.34)**	**0.019**

OR, odds ratio; CI, confidence internal.

^a^Adjusted for education, occupation, smoking, passive smoking, age, pre-pregnancy BMI, abortion history, PCOS, GDM history, macrosomia, family history of diabetes and family history of gestational diabetes. Bold values indicate statistically significant values.

**TABLE 3:** Odds ratios (OR) and 95% confidence intervals (CI) of GDM by quartile of B vitamins.

Nutrients	GDM/pregnancy	Crude OR^a^ (95% CI)	Adjusted OR^a^ (95% CI)	*P*_trend_
**Dietary B6 (mg/day)**				**<0.001**
Q1 (0.05–0.65)	134/817	1.00	1.00	
Q2 (0.66–1.06)	124/807	0.93 (0.71–1.21)	1.16 (0.81–1.66)	
Q3 (1.07–1.52)	160/824	1.23 (0.95–1.58)	1.56 (1.10–2.22)	
Q4 (1.53–3.32)	167/804	**1.34 (1.04**–**1.72)**	**1.60 (1.13**–**2.27)**	
**Supplement B6 (mg/day)**				0.228
Q1 (0–0.27)	151/918	1.00	1.00	
Q2 (0.28–1.98)	142/779	1.13 (0.88–1.46)	1.17 (0.88–1.55)	
Q3 (1.99–2.60)	169/896	1.18 (0.93–1.50)	1.05 (0.80–1.38)	
Q4 (2.61–9.30)	123/659	1.17 (0.89–1.52)	1.26 (0.94–1.68)	
**Total B6 (mg/day)**				**0.029**
Q1 (0.14–1.59)	131/810	1.00	1.00	
Q2 (1.60–2.98)	142/816	1.09 (0.84–1.42)	0.97 (0.71–1.33)	
Q3 (2.99–3.78)	137/806	1.06 (0.82–1.38)	1.04 (0.78–1.40)	
Q4 (3.79–10.50)	175/820	**1.41 (1.09**–**1.81)**	**1.33 (1.00**–**1.77)**	
**Dietary B12 (μ g/day)**				**<0.001**
Q1 (0.05–0.63)	128/819	1.00	1.00	
Q2 (0.64–1.69)	140/816	1.12 (0.86–1.45)	1.36 (0.96–1.94)	
Q3 (1.70–3.01)	134/809	1.07 (0.82–1.39)	1.34 (0.94–1.90)	
Q4 (3.02–9.80)	183/808	**1.58 (1.23**–**2.03)**	**1.88 (1.34**–**2.64)**	
**Supplement B12 (μ g/day)**				0.076
Q1 (0–0.41)	149/919	1.00	1.00	
Q2 (0.42–4.00)	160/953	1.17 (0.95–1.44)	1.17 (0.92–1.48)	
Q3 (4.01–5.00)	158/736	1.48 (0.66–3.30)	1.00 (0.37–2.70)	
Q4 (5.01–9.33)	118/644	1.27 (0.93–1.75)	1.32 (0.93–1.87)	
**Total B12 (μ g/day)**				**0.002**
Q1 (0.13–2.68)	125/812	1.00	1.00	
Q2 (2.697–4.87)	138/809	1.13 (0.87–1.47)	1.03 (0.75–1.41)	
Q3 (4.88–6.59)	141/814	1.15 (0.89–1.50)	1.09 (0.81–1.47)	
Q4 (6.60–14.84)	181/817	**1.56 (1.22**–**2.01)**	**1.52 (1.14**–**2.02)**	
**Dietary folate (μ g/day)**				**<0.001**
Q1 (5–87)	139/831	1.00	1.00	
Q2 (88–178)	122/808	0.89 (0.68–1.15)	1.10 (0.78–1.57)	
Q3 (179–284)	147/808	1.11 (0.86–1.43)	1.33 (0.94–1.87)	
Q4 (285–1,396)	177/805	**1.40 (1.10**–**1.80)**	**1.66 (1.19**–**2.32)**	
**Supplement folate (μ g/day)**				**0.033**
<400	164/977	1.00	1.00	
400–800	238/1,399	1.02 (0.82–1.26)	0.93 (0.73–1.20)	
>800	183/876	**1.31 (1.04**–**1.65)**	**1.30 (1.01**–**1.68)**	
**Total folate (μ g/day)**				**0.027**
Q1 (14–409)	133/812	1.00	1.00	
Q2 (410–850)	143/814	1.09 (0.84–1.41)	0.97 (0.71–1.36)	
Q3 (851–1,499)	139/813	1.05 (0.81–1.37)	1.02 (0.77–1.36)	
Q4 (1,500–2,778)	170/813	**1.35 (1.05**–**1.74)**	**1.35 (1.02**–**1.77)**	

*P*_trend_, Tests for trend were performed using the median of the interval for each quartile. Reference to control group of women who was no-GDM.

^a^Adjusted for education, occupation, smoking, passive smoking, age, pre-pregnancy BMI, abortion history, PCOS, GDM history, macrosomia, family history of diabetes and family history of gestational diabetes. Bold values indicate statistically significant values.

**TABLE 4:** Association of ART with maternal gestational diabetes mellitus, stratified by supplemental folate.

	Spontaneous/ART	Crude OR^a^ (95% CI)	Adjusted OR^a^ (95% CI)	*P*
**Supplemental folate (μg/day)**				
<400	903/74	1.29 (0.71–2.33)	1.07 (0.54–2.14)	0.840
400–800	1,263/136	1.65 (1.09–2.52)	1.17 (0.68–2.00)	0.575
>800	753/123	2.15 (1.42–3.27)	**1.62 (1.39**–**3.39)**	**0.043**

OR, odds ratio; CI, confidence internal. Reference to control group of women who conceived spontaneously in the respective supplemental folate group.

^a^All models adjusted for adjusted education, occupation, smoking, passive smoking, age, pre-pregnancy BMI, abortion history, PCOS, GDM history, macrosomia, dietary vitamin B6, dietary vitamin B12, family history of diabetes and family history of gestational diabetes. Bold values indicate statistically significant values.

**TABLE 5:** Association of ART with maternal glucose concentrations, stratified by supplemental folate.

	Fasting glucose^b^ (mmol/L)		OGTT 1-h glucose^b^ (mmol/L)		OGTT 2-h glucose^b^ (mmol/L)	
	β ^a^ (95% CI)	*P*	β ^a^ (95% CI)	*P*	β^a^ (95% CI)	*P*
**Supplemental folate (μg/day)**						
<400	0.03 (–0.15 to 0.21)	0.750	0.13 (–0.45 to 0.70)	0.667	0.14 (–0.31 to 0.60)	0.529
400–800	–0.01 (–0.14 to 0.11)	0.826	-0.09 (–0.51 to 0.33)	0.668	0.02 (–0.34 to 0.37)	0.935
>800	0.07 (–0.05 to 0.17)	0.247	**0.76 (0.39–1.13)**	**<0.001**	**0.60 (0.29–0.92)**	**<0.001**

β, regression coefficients; CI, confidence internal. Reference to control group of women who conceived spontaneous conception.

^a^All models adjusted for adjusted education, occupation, smoking, passive smoking, age, pre-pregnancy BMI, abortion history, PCOS, GDM history, macrosomia, dietary vitamin B6, dietary vitamin B12, family history of diabetes and family history of gestational diabetes.

^b^Among all the 2,258 women of which 2,031 were spontaneous conception and 227 were receiving ART. Bold values indicate statistically significant values.

### Exposure and outcome measures

ART involves the *in vitro* handling of human oocytes and sperm, or embryos, intended to bring pregnancy (21). At enrollment, women responded to a semiquantitative food frequency questionnaire (FFQ) (22), reporting the frequency at which they consumed a specific portion of each of the 25 food or food group items during the past 6 months. They also reported their use of dietary supplements, including the brand, dose, and frequency. Intake of dietary folate, vitamin B6, and vitamin B12 from diet were calculated by multiplying the frequency of consumption by the nutrient content of a serving size determined from the food composition values available from the China Food Composition Tables (6th edition) (23). Total folate, vitamin B6 and B12, multivitamins and supplements and dietary sources were considered (24). FFQ was previously validated, which provides valid estimates of vitamins B6 and B12 intake with correlation coefficients between the FFQ and 1-week diet records of 0.58 for vitamin B6 (25), and 0.56 for vitamin B12 (26) intake. Furthermore, folate intake correlates well with prospectively collected diet records of 0.71 (27).

All participants were screened for GDM using a 75 g at 24–28 weeks gestation, according to the Ministry of Health (MOH) of China’s Diagnostic Criteria for Gestational Diabetes Mellitus (WS311-2011) (28). MOH criterion cut-off values were consistent with the Consensus Panel recommendations of the International Association of Diabetes and Pregnancy Study Groups (29). In summary, GDM could be diagnosed if any of the following 75 g OGTT values were met or exceeded: fasting plasma glucose (FPG) ≥ 5.1 mmol/L, 1-h ≥ 10.0 mmol/L, or 2-h ≥ 8.5 mmol/L.

### Covariates

We relied on self-reported data on maternal characteristics that could influence B vitamins intake and maternal risk of GDM. All multivariable models adjusted for maternal education (secondary school or less, high school, college/university, postgraduate and above), occupation (no, yes), women smoking and passive smoking during preconception to the first trimester (no, yes), age, pre-pregnancy BMI, and abortion history (no, yes), PCOS history (no, yes), GDM history (no, yes), and macrosomia (≥ 4 kg) (no, yes) are part of the personal medical history. Also, the family history of the disease includes diabetes and gestational diabetes (no, yes).

*E*-values were evaluated to estimate the association between ART and GDM risk so that the resistance of this study’s results to unmeasured confounders could be assessed. The *E*-value is a sensitivity analysis representing the minimum strength of association on the risk ratio scale that an unmeasured confounder would need to have with both the exposure and the outcome to explain a specific exposure-outcome association (30) fully.

### Statistical analysis

Based on the way of fertilization, the study population was divided into two groups as indicated in Figure 1. As shown in Table 1, continuous and categorical maternal characteristics between spontaneous conception and ART women were compared. The Kolmogorov-Smirnov test and histograms were used to examine the distribution of continuous variables. Continuous variables having a normal distribution are displayed as means and standard deviations (mean ± SD). The median and interquartile range (IQR) are displayed for variables that significantly deviate from the normal distribution. Categorical variables are presented as frequencies with percentages. For regularly distributed continuous variables, differences were assessed using the Student’s *t*-test. The Mann-Whitney *U*-test examined the differences between continuous variables with non-normal distribution. In order to assess the differences between categorical variables, the chi-square test was used.

Covariates in the multivariate models were selected based on a significant association at alpha < 0.05 level in bivariate models. The odds ratio (OR) and 95% confidence interval (CI) of GDM in ART vs. spontaneous conception were estimated using multivariable binomial logistic regression models. Multivariable linear regression models reporting regression coefficient (β) of fasting, 1-h OGTT glucose, and 2-h OGTT glucose levels between the two conception groups were used. All multivariable models were adjusted for maternal age, education, occupation, smoking, passive smoking, pre-pregnancy BMI, abortion history, PCOS, GDM history, macrosomia, family history of diabetes and family history of gestational diabetes.

Based on the distribution, the intake of folate, vitamin B6, and vitamin B12 were divided into quartiles. US Preventive Services Task Force (USPSTF) and WHO advise taking a daily folate supplement of 400–800 μg/day for all women who are consider becoming pregnant or are capable of becoming pregnant (31, 32). Furthermore, recent cohort studies in China indicated that taking folate supplements at doses higher than 800 μg/day increased the risk of maternal GDM and hypertension (33, 34). The consumption of folate supplements in this study has been categorized as inadequate (< 400 μg/day), adequate (400–800 μg/day), and excessive (> 800 μg/day). ORs and 95% CIs were estimated for all three nutrients. Moreover, linear trends of GDM risk across the three nutrient intake categories were explored, by employing the median intake of each category as a continuous variable when fitting the models.

A multiplicative interaction term was included in the multivariable logistic and linear regression models to test our hypothesis that the three nutrients will change the incidence rate of GDM in ART women. However, the interaction between supplemental folate and ART on GDM and OGTT blood glucose levels is statistically significant (*P* for interaction < 0.05). The association between ART and GDM and OGTT blood glucose levels stratifying by supplemental folate was further evaluated: inadequate folate < 400 μg/day, adequate folate (400–800) μg/day, and excessive folate > 800 μg/day. No other effect modifiers were proposed, and no other interactions were investigated. All the data were analyzed with SPSS 22.0 software.

## Results

Between 2019 and 2022, 3,252 eligible singleton pregnancies were among 3,980 women. 333 (10.24%) of these women underwent ART, while 2,919 (89.76%) conceived spontaneously. Table 1 shows the characteristics of the study population. Distinctions exist between the ART and spontaneous conception groups. Women in the ART groups were likelier to have lower education level, nulliparous, no occupation and higher BMI. Regarding nutrient intake, the ART group possibly received higher doses of supplemental B6, total B6, supplemental B12, total B12, and total folate. Similarly, OGTT 1-h and OGTT 2-h blood glucose levels were greater than the spontaneous conception group (Table 1).

As shown in Table 2, in the unadjusted analysis, women who conceived ART exhibited almost double the odds of GDM than the spontaneous conception women (OR = 1.78, 95% CI: 1.37–2.31). After adjusting for maternal education, occupation, smoking, passive smoking, age, pre-pregnancy BMI, abortion history, PCOS, GDM history, macrosomia history, and family history of diabetes and gestational diabetes, the odds of GDM remained significantly higher in the ART group (aOR = 1.59, 95% CI: 1.08–2.34).

Fasting blood glucose levels between the ART and the spontaneous conception groups were not statistically different adjusting for variables. Neither were the regression coefficients in the multivariable linear regression models. However, OGTT 1-h and OGTT 2-h blood glucose levels were significantly higher in the ART group before adjustment, continuing the same trend after adjustment for covariates. The regression coefficients (β) of 1-h blood glucose (β = 0.43, 95% CI: 0.19–0.68) and 2-h blood glucose levels (β = 0.40, 95% CI: 0.19–0.60) in multiple linear regression are also statistically significant (Figure 2).

Comparing the highest to the lowest quartile, a significant relationship between dietary vitamin B6 and GDM (aOR = 1.60, 95% CI: 1.13–2.27) has been found. Similar results were observed for total vitamin B6 (aOR = 1.33, 95% CI: 1.00–1.77), including supplemental vitamin B6 sources. Both dietary vitamin B12 and total vitamin B12 exhibited a significant correlation with GDM in the highest quartile (aOR = 1.88, 95% CI: 1.34–2.64, and aOR = 1.52, 95% CI: 1.14–2.02) (*P*_*trend*_ < 0.05). The association between total vitamin B6 and GDM risk was entirely driven by dietary vitamin B6, similar to vitamin B12. Dietary folate and total folate intake were positively associated with GDM risk. The aORs of GDM for dietary folate and total folate intake were 1.66 (95% CI: 1.19–2.32) and 1.35 (95% CI: 1.02–1.77) (*P*_*trend*_ < 0.05). The aORs of GDM by supplemental folate intake were 0.93 (95% CI: 0.73–1.20) for 400–800 μg/day and 1.30 (95% CI: 1.01–1.68) for > 800 μg/day compared to intake < 400 μg/day (*P*_*trend*_ = 0.033) (Table 3).

Since only the interaction between supplemental folate and the ART on GDM and OGTT blood glucose levels was statistically significant (*P* for interaction < 0.05), we further stratified by supplemental folate: inadequate folate < 400 μg/day, adequate folate (400–800) μg/day, and excessive folate > 800 μg/day, and found that women with ART and excessive supplemental folate (> 800 μg/day) showed a slightly higher risk of developing GDM than women with spontaneous conception (aOR = 1.62, 95% CI: 1.39–3.39) (Table 4). Moreover, the increase in 1-h OGTT blood glucose risk (β = 0.76, 95% CI: 0.39–1.13) as well as 2-h OGTT blood glucose risk (β = 0.60, 95% CI: 0.29–0.92), were statistically significant only in women with excessive supplemental folate (> 800 μg/day) and ART (Table 5). All multivariable models adjusted for maternal age, education, occupation, smoking, passive smoking, pre-pregnancy BMI, abortion history, PCOS, GDM history, macrosomia, dietary vitamin B6, dietary vitamin B12, family history of diabetes and family history of gestational diabetes.

## Discussion

This study demonstrated that women-conceived ART appears to be a risk factor for GDM and elevated blood glucose levels. We also found that ART was linked with GDM and higher blood glucose concentrations 1- and 2-h after OGTT in the 24–28 weeks, especially in women using excessive supplemental folate (> 800 μg/day).

Ashrafi et al. carried out a similar study and found that women who underwent ART had a higher risk of GDM than those who were spontaneously pregnant. However, they did not observe significant differences in blood glucose levels. The primary cause of the discrepancy is the various GDM judging standards. Their study followed the guidelines set forth by the American Diabetes Association (35), which called for two-step screening pregnant women at 24–28 weeks of gestation with a 50 g, 1-h oral glucose challenge test. If the screening test’s results were abnormal (glucose ≥ 7.8 mmol/l or 140 mg/dl), a 100 g 3-h oral glucose tolerance test (OGTT) was then carried out in the 1–2 weeks that followed. Our study used one-step screening (i.e., a glucose-tolerance test in which the blood glucose level was obtained after the oral administration of a 75 g glucose load in the fasting state). It has been indicated that the two-step strategy led to lower diagnoses of GDM than the one-step approach (35). Another reason is that their sample size is relatively small, only including 360 women (36). Cai et al. found that IVF was associated with an increase in fasting blood glucose concentration in the second and third trimesters of pregnancy 2-h after OGTT, but only in women with a BMI of 25 kg/m^2^ or higher (37). However, this study lacked a 1-h post-OGTT plasma glucose sample. Szymanska et al. observed an increase in fasting blood glucose levels during the first trimester of an IVF pregnancy but no increase in blood glucose levels during the second trimester. However, the study was limited to women with GDM (38).

Additionally, the results of the OGTT blood glucose test were examined in our study, and it was shown that women underwent ART would experience an increase in 1-h and 2-h blood glucose levels with no effect on fasting blood glucose. Gestational abnormal glucose metabolism is associated with a remarkably increased risk of adverse perinatal outcomes, even when levels are below GDM thresholds (39–41), and it is worth noting to observe the magnitude of these effects.

According to previous research, the risk of GDM may be increased by the relative deficiency of vitamin B6 and the comparatively low intake of vitamin B12 (42, 43). However, a positive correlation has been found between total vitamin B6 and total vitamin B12 in the highest quartile and the risk of GDM. This may be due to the fact that the supplements’ contents were primarily the focus of the earlier research. However, our study’s association between total vitamins B6 and B12 on GDM risk was entirely driven by dietary vitamins B6 and B12.

Both dietary folate and supplemental folate are statistically associated with GDM. Nevertheless, 10 years cohort study showed no statistically significant association between dietary folate before pregnancy and the prevalence of GDM (44). Given that sufficient dietary folate (> 400 μg/day) is expected to be rare in our sample, only 13.10% of our sample exhibited dietary folate intakes meet the recommended daily allowance (45). It is predicted that different associations between dietary folate and GDM in the study may be due to the different folate intakes of various populations. According to the USPSTF and WHO recommendations (31, 32), in this investigation, supplemental folate of > 800 μg/day is considered to be excessive, revealing that excessive folate supplementation would increase the incidence rate of GDM. Consistent with our study results, a cohort conducted in China showed that periconceptional folate supplement use of more than 800 μg/day from pre-pregnancy through mid-pregnancy was related to elevated GDM risk (34). The existing research on the relationship between B vitamins (folate, vitamin b6, and vitamin b12) and GDM is still controversial, more in-depth studies across multiple populations are needed to verify these results.

Previous studies have also considered the folate impact on women with ART, showing that folate can improve total and mature oocyte counts (46), embryo quality (47), pregnancy rates (48) and increase the likelihood of embryo survival (19, 49) after infertility treatment. However, these studies did not consider the effect of folate supplementation on perinatal diseases in pregnant women received ART. Numerous studies have demonstrated that folate supplementation, particularly excessive supplemental folate (> 800 μg/day), can alter the incidence of GDM in spontaneous conception women (34, 44, 50). Our research enriches fields related to ART and folate supplementation and showed that pre-pregnancy to first trimester folate supplements could modify the effect of ART on GDM risk and blood glucose levels.

The strengths of our study include a unique design and aim, specifically evaluating whether women on B vitamins exhibit a higher risk of GDM after ART. Moreover, using of a prospective cohort, which reduces the effects of selection or recall bias. To avoid information bias, women with hypertension or diabetes mellitus, as well as major influences of GDM were excluded. The *E*-value for the association between the women who underwent ART and GDM risk was 1.83, suggesting that a potential confounder must have relatively strong associations with both women who underwent ART and GDM risk to fully explain the association between supplemental folate intake and GDM risk.

This study has some limitations. The plasma homocysteine levels of B vitamins in pregnant women were not measured to support our conclusions. As a result, there is a possibility of misclassification. However, significant efforts were made to ensure reliable vitamin B data were collected on time by trained medical personnel with meticulous follow-up. Furthermore, self-reported folate from supplements was correlated with plasma folate and was regarded as a reliable indicator of folate exposure (51). Also, a relatively small sample size limits our ability to investigate the relationship between B vitamins and ART at different levels, and the generalizability of the findings to other regions needs further validation. Therefore, it is necessary to perform such study on a larger population.

## Conclusion

ART appears to be an independent risk factor for GDM and elevated blood glucose levels. Our findings reinforce the need to advise women contemplating ART to avoid excessive folate supplements (>800 μg/day) to reduce their risk of GDM and hyperglycemia-related adverse outcomes. Women who seek fertility therapy should be counseled about this potential risk, and efforts should be taken to regulate the dose of folate supplementation throughout pregnancy. Pregnant women who underwent ART should have their health care during pregnancy, focusing more on blood glucose fluctuations.

## Data availability statement

The raw data supporting the conclusions of this article will be made available by the authors, without undue reservation.

## Ethics statement

Ethical approval for this study has been obtained from the Ethics Committee of Qingdao Women’s and Children’s Hospital (Number: 019–2019-FEKY). The patients/participants provided their written informed consent to participate in this study.

## Author contributions

ML and HW conceived, designed, and supervised the cohort study. YW and YC conceptualized idea for the manuscript. ML, HW, and XD were involved in design of questionnaire and protocol as well as supervision of data collection, analyzed, and interpreted the data. GL contributed to the preparation of the manuscript. All authors critically revised the article for intellectual and scientific content and approved this submission for publication.
